# Supplementary material for: Highly Transparent Red Organic Light-Emitting Diodes with AZO/Ag/AZO Multilayer Electrode
Source: Micromachines (Basel). 2024 Jan 18;15(1):146. doi: 10.3390/mi15010146 (PMC10818506; doi:10.3390/mi15010146)
Supplement: Supplementary file 1 [file micromachines-15-00146-s001.zip › micromachines-2832578-supplementary.pdf]

micromachines

MDPI

Article

Highly Transparent Red Organic Light-Emitting Diodes with AZO/Ag/AZO Multilayer Electrode

Dongwoon Lee [1,†], Min Seok Song [1,†], Yong Hyeok Seo [1], Won Woo Lee [1], Young Woo Kim [1], Minseong Park [2], Ye Ji Shin [2], Sang Jik Kwon [1,] Yongmin Jeon [2,*], and Eou-Sik Cho [1,*]

[1] Department of Electronics Engineering, Gachon University, Seongnam 13120, Republic of Korea.
[2] Department of Biomedical Engineering, Gachon University, Seongnam 13120, Republic of Korea.
* Correspondence: yongmin@gachon.ac.kr (Yongmin Jeon), es.cho@gachon.ac.kr (Eou-Sik Cho)
† These authors contributed equally to this work.

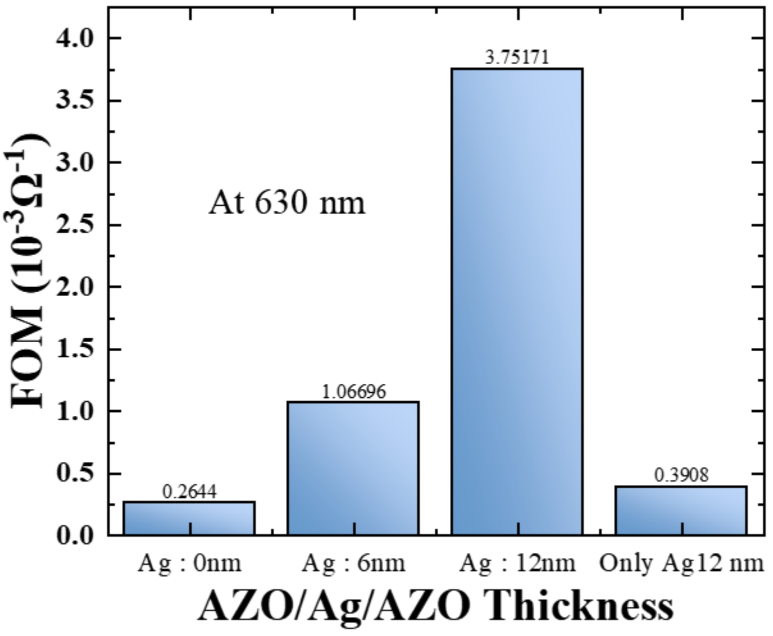

Figure S1. FOM value of the AZO/Ag/AZO at a wavelength of 630 nm according to the Ag thickness in Figure 1d.

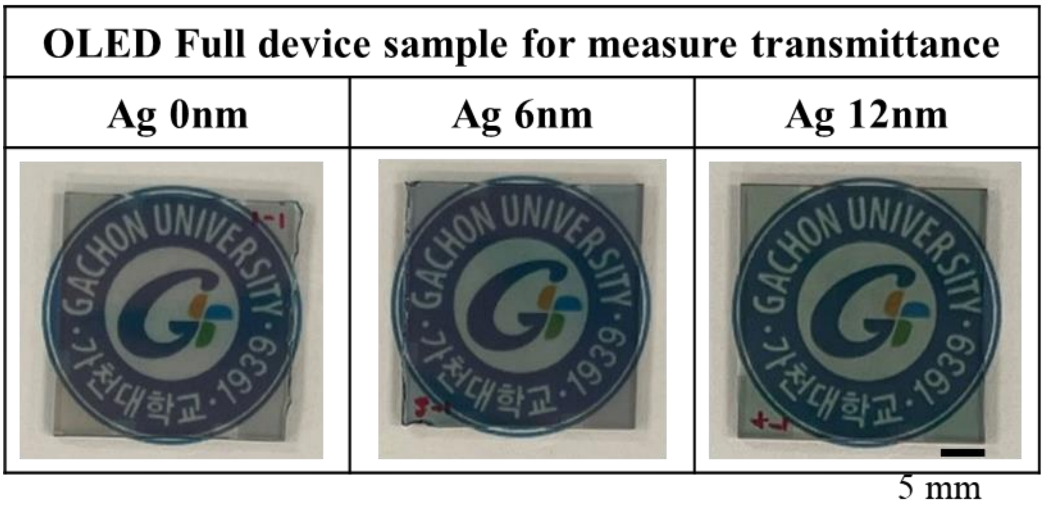

Figure S2. Optical imagery of the transparent red OLED for different Ag thickness in the AZO/Ag/AZO anode.

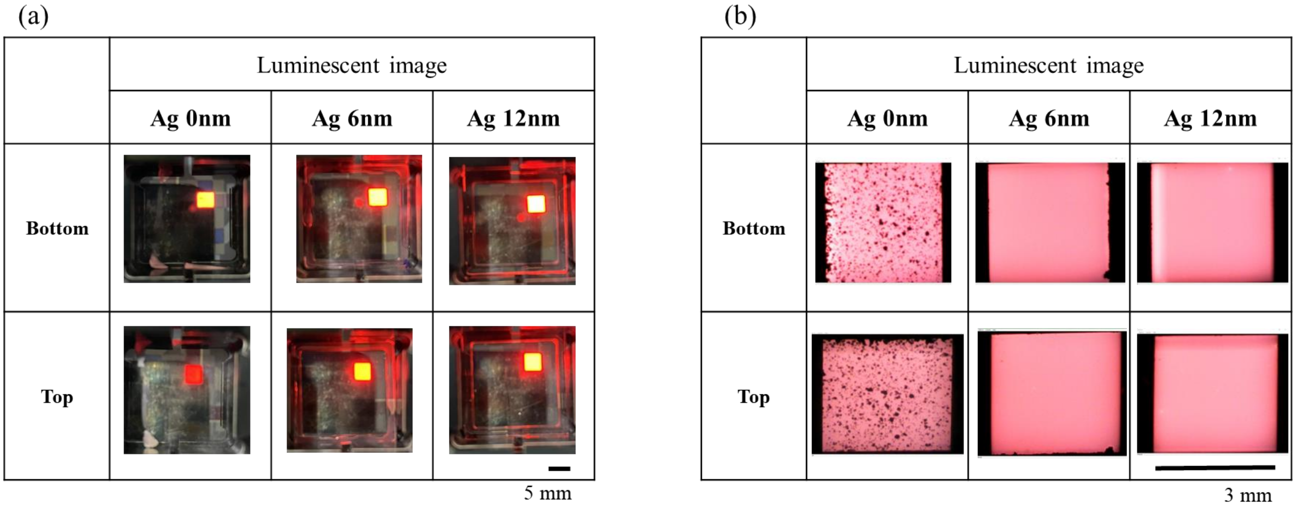

Figure S3. Luminescent imagery of the fabricated red OLED in both the top and bottom directions: (a) normal size, and (b) magnified size.

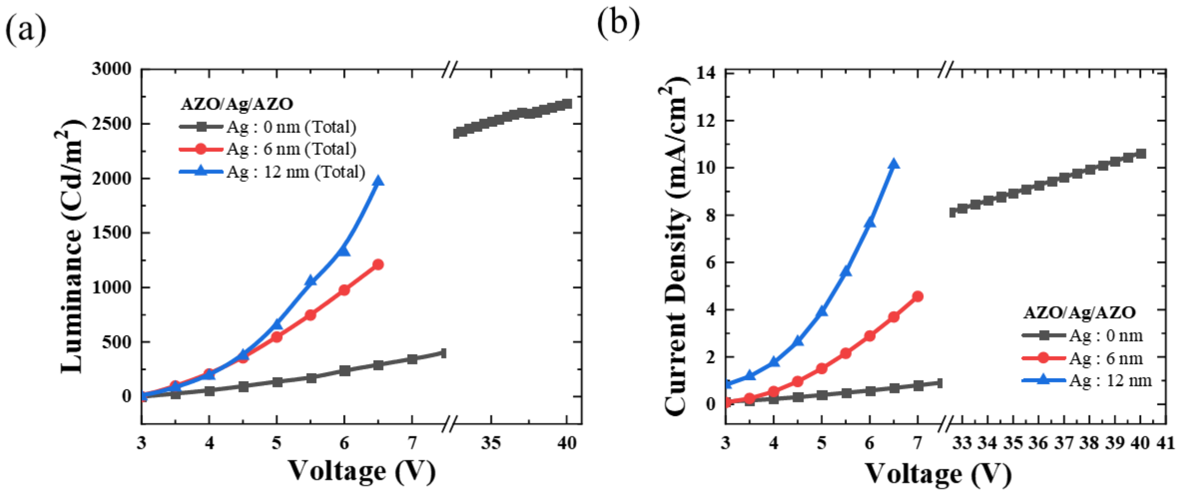

Figure S4. Analysis of the total emission characteristics of the fabricated transparent red OLED with different Ag thickness in the AZO/Ag/AZO anode: (a) The luminance–voltage characteristics of the fabricated red OLED for different Ag thickness in the AZO/Ag/AZO anode; (b) the current density – voltage characteristics of the fabricated red OLED for different Ag thickness in the AZO/Ag/AZO anode.

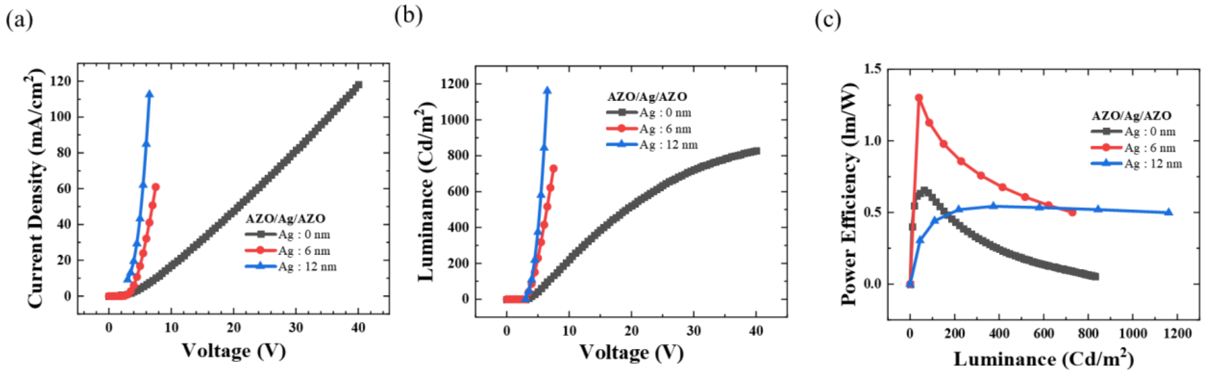

Figure S5. Analysis of the total emission characteristics of the fabricated transparent red OLED with different Ag thickness in the AZO/Ag/AZO anode: (a) the current density – voltage characteristics of the fabricated red OLED for different Ag thickness in the AZO/Ag/AZO anode; (b) The luminance–voltage characteristics of the fabricated red OLED for different Ag thickness in the AZO/Ag/AZO anode; and (c) the power efficiency – luminance characteristics of the fabricated red OLED for different Ag thickness in the AZO/Ag/AZO anode.

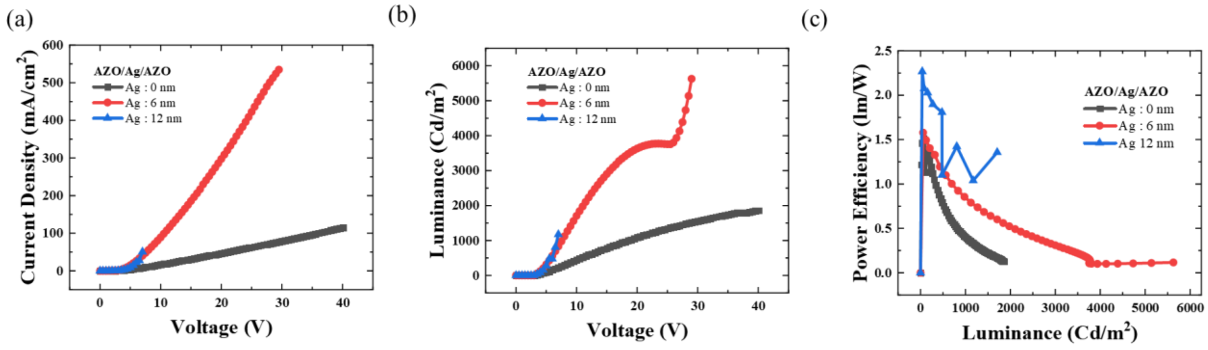

Figure S6. Analysis of the bottom emission characteristics of the fabricated transparent red OLED with different Ag thickness in the AZO/Ag/AZO anode: (a) the current density – voltage characteristics of the fabricated red OLED for different Ag thickness in the AZO/Ag/AZO anode; (b) the luminance–voltage characteristics of the fabricated red OLED for different Ag thickness in the AZO/Ag/AZO anode; and (c) the power efficiency – luminance characteristics of the fabricated red OLED for different Ag thickness in the AZO/Ag/AZO anode.
